# Short- and long-term mortality of subarachnoid hemorrhage according to hospital volume and severity using a nationwide multicenter registry study

**DOI:** 10.3389/fneur.2022.952794

**Published:** 2022-08-05

**Authors:** Sang-Won Park, Ji Young Lee, Nam Hun Heo, James Jisu Han, Eun Chae Lee, Dong-Yong Hong, Dong-Hun Lee, Man Ryul Lee, Jae Sang Oh

**Affiliations:** ^1^Department of Neurosurgery, College of Medicine, Cheonan Hospital, Soonchunhyang University, Cheonan, South Korea; ^2^Department of Molecular Biophysics and Biochemistry, Yale University, New Haven, CT, United States; ^3^Soonchunhyang Institute of Medi-Bio Science (SIMS), Soonchunhyang University, Cheonan, South Korea

**Keywords:** subarachnoid hemorrhage, long-term outcomes, short-term outcomes, mortality, hospital volume

## Abstract

**Introduction:**

Recent improvements in treatment for subarachnoid hemorrhage (SAH) have decreased the mortality rates; however, the outcomes of SAH management are dependent on many other factors. In this study, we used nationwide, large-scale, observational data to investigate short- and long-term mortality rates after SAH treatment and the influence of patient severity and hospital volume.

**Patients and methods:**

We selected patients with SAH treated with clipping and coiling from the South Korean Acute Stroke Assessment Registry. High- and low-volume hospitals performed ≥20 clipping and coiling procedures and <20 clipping and coiling procedures per year, respectively. Short- and long-term mortality were tracked using data from the Health Insurance Review and Assessment Service.

**Results:**

Among 2,634 patients treated using clipping and coiling, 1,544 (58.6%) and 1,090 (41.4%) were hospitalized in high- and low-volume hospitals, respectively, and 910 (34.5%) and 1,724 (65.5%) were treated with clipping and coiling, respectively. Mortality rates were 13.5, 14.4, 15.2, and 16.1% at 3 months, 1, 2, and 4 years, respectively. High-volume hospitals had a significantly lower 3-month mortality rate. Patients with mild clinical status had a significantly lower 3-month mortality rate in high-volume hospitals than in low-volume hospitals. Patients with severe clinical status had significantly lower 1- and 2-year mortality rates in high-volume hospitals than in low-volume hospitals.

**Conclusion:**

Short- and long-term mortality in patients with SAH differed according to hospital volume. In the modern endovascular era, clipping and coiling can lead to better outcomes in facilities with high stroke-care capabilities.

## Introduction

Subarachnoid hemorrhage (SAH) accounts for 8.9% of all stroke cases in Korea (1). However, its 30-day mortality rate is 44%, and 10–15% of patients die before arriving at the hospital (2). The development of new devices and improvements in coiling techniques to treat ruptured intracranial aneurysms has reduced mortality (3–5). However, most reports on the prognosis of SAH have come from high-volume hospitals with experienced neurosurgeons, special care units, and nurses (6, 7). Moreover, the outcomes of SAH are dependent on patient-level factors, such as age, sex, clinical status, degree of brain edema, and comorbidities, as well as hospital-level factors, such as experienced and skilled neurosurgeons, hospital capacity, stroke unit, and the number of doctors and nurses. Because these results cannot be generalized to the real-world clinical environment; research using nationwide, large-scale, observational data is warranted to investigate the prognosis after SAH treatment and study the influence of patient severity and hospital volume. In this study, we investigated short- and long-term mortality after SAH treatment according to hospital volume using nationwide registry data from Korea.

## Patients and methods

### Data and study population

In 2013, South Korea established the Acute Stroke Assessment Registry (ASAR), a nationwide, pre-collected database from 248 preselected hospitals, to assess stroke quality and improve the outcomes of patients with stroke. The Korean healthcare and medical insurance system cover the entire Korean population, and the records can be anonymously linked to the Health Insurance Review and Assessment Service (HIRA) database using encrypted personal identification numbers so that researchers can determine survival outcomes among patients with SAH. Skilled medical staff from preselected hospitals are responsible for collecting detailed data from patients visiting the emergency department for stroke. Based on the data, the HIRA also provides feedback and grades for each hospital.

Patient data were protected and kept anonymous throughout the study period. The present study was conducted with the HIRA under the Joint Project on Quality Assessment Research (M20210128971). Data collected by ASAR from March to May 2013, June to August 2014, June to December 2016, and June to December 2018 were linked with the HIRA database. Patients with hemorrhagic stroke were defined as those with hemorrhagic stroke as the primary disease (*International Classification of Disease, 10th version* [*ICD-10*]: I60) and who were hospitalized in the emergency department within 7 days of symptom onset. Patients with traumatic injuries were excluded from the study to rule out trauma-induced hemorrhagic stroke. To reduce confusion due to mixed cases, we limited the dataset to patients with their first hemorrhagic stroke who had not been hospitalized for primary or secondary disease related to hemorrhagic stroke in the past year. The surgeries were limited to aneurysm clipping and endovascular coiling, which are the major surgical techniques used to treat SAH. We analyzed acute stroke data from these patients as well as data obtained during the follow-up period. All patients included in this study were monitored until April 15, 2021. Mortality was identified based on the Statistics Korea data.

To investigate the change in the SAH treatment trend, the annual change in aneurysm clipping and endovascular coiling was investigated. In addition, the change in the treatment ratio according to the classification of high-volume hospitals and low-volume hospitals was also investigated.

### Study population

**Figure 1:**
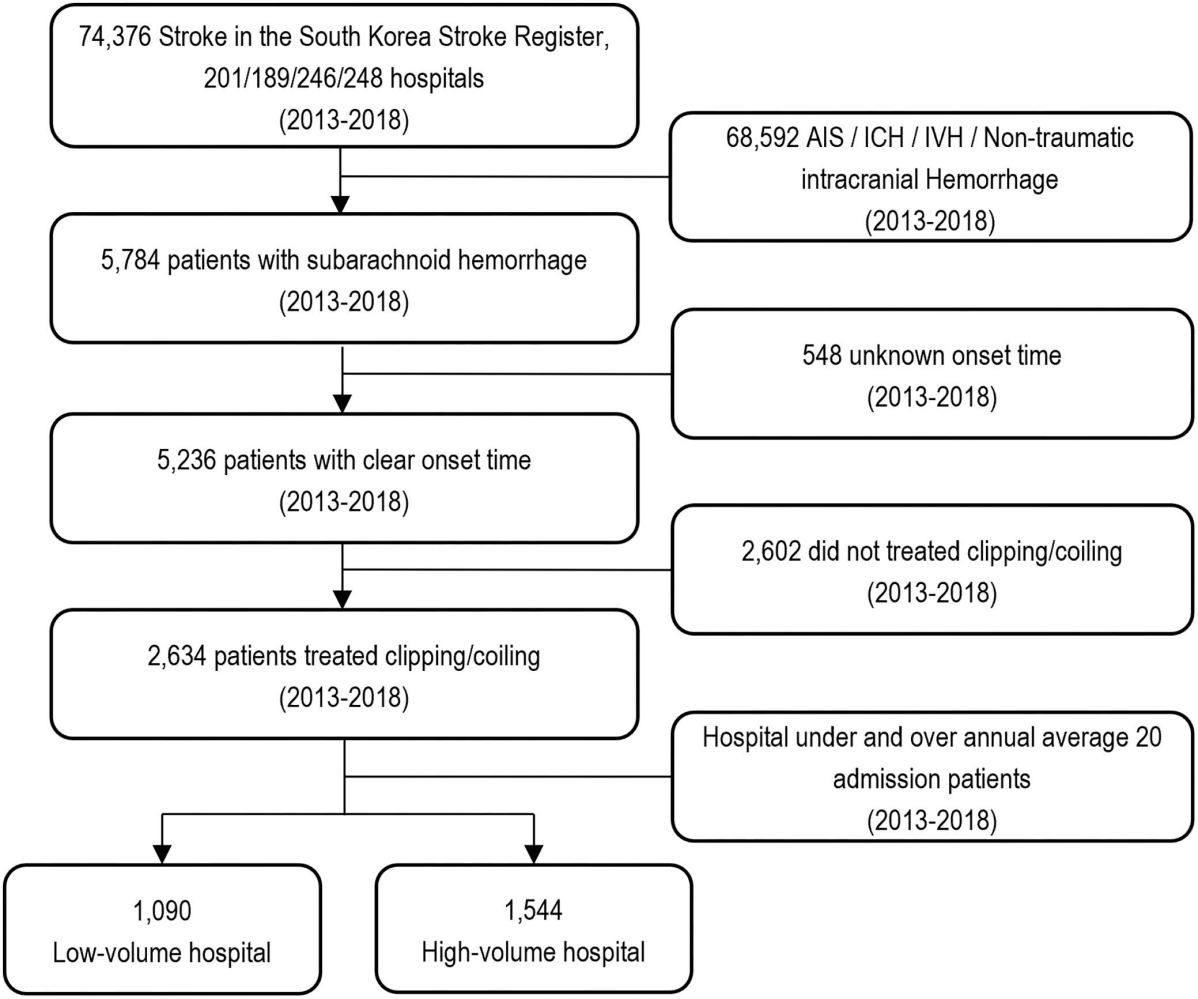
Flow chart of the study population and patients with subarachnoid hemorrhage according to hospital volume.

Over 18 months, 74,376 patients with acute stroke were admitted to the emergency department. Of these, 5,784 (7.8%) patients experienced an acute hemorrhagic stroke. Patients who received conservative treatment for SAH were excluded to facilitate analysis and comparison of hospital volume and treatment outcomes. A total of 548 (9.5%) patients were excluded because the symptom onset time was not recorded. Of the 5,236 patients, 2,602 (49.7%) were excluded because they did not undergo clipping or coiling. In total, 2,634 (50.3%) patients were treated with clipping and coiling (Figure 1).

In our previous research, hospital volume was divided annually by 20 cases of clipping and coiling procedures to avoid intentional cutoff level bias (8, 9). Since this criterion was significant in categorizing the volume of the hospital, we divided patients into two groups by 20 cases/year, namely, high- (≥20 cases/year) and low-volume hospitals (<20 cases/year). We determined a cutoff Glasgow Coma Scale (GCS) score of 9 to classify the severity of clinical status in patients with SAH (10). We categorized the severity of clinical status according to the National Institute of Health Stroke Scale (NIHSS) and the GCS on admission as follows: (1) severe clinical status (NIHSS ≥ 16 or GCS ≤ 9) and (2) mild clinical status (NIHSS ≤ 15 or GCS ≥ 10). Economic status was determined based on whether the patient received health insurance or medical aid. The patients were also divided into current smokers, ex-smokers, and non-smokers. The Charlson Comorbidity Index (CCI) was determined using the *ICD-10* code and was used to divide patients into four groups: 0, 1, 2, and ≥3. Data on the use of emergency medical services (EMS) before arrival at the hospital, onset-to-door time, and door-to-image time were also collected. Functional outcome at discharge was defined as good or poor; a good functional outcome was defined as 75–99 points on the Korean Version of Modified Barthel Index (K-MBI), the Modified Barthel Index (MBI), or the Barthel Index (BI) (11); 90 or higher on the Functional Independence Measure (FIM) (12); 2 or lower on the modified Rankin Scale (mRS) (13); and 5 on the Glasgow Outcome Scale (GOS) (14). Mortality after admission was divided into four categories: within 3 months, 1, 2, and 4 years.

### Hospital costs

Insurance coverage was factored into hospital costs, and all amounts, including drug prices, were extracted from the ASAR. All direct medical costs for beds, staff, examinations, medications (including surgical procedures), rehabilitation, and other minor miscellaneous expenses, e.g., commissions, were calculated. The total amount of claim care benefit expenses and drug details of the medical institutions of patients with SAH as the main disease was defined as the hospital cost. The cost was calculated in Korean Won (KRW and then converted to United States dollar (USD $) using an exchange rate of USD$1= KRW 808 according to the purchasing power parties (PPP) of 2022 on the Organization for Economic Co-operation and Development (15).

### Statistical analysis

Categorical variables between high- and low-volume hospitals were compared using the χ^2^ test. Descriptive data are expressed as percentages. The primary outcomes of this study were short-term (3-month, 1-year) and long-term (2, 4-year) mortality after SAH onset. Kaplan–Meier survival estimates (KM) and the differences between survival curves were tested using the log-rank test stratified by matched sets. The Cox proportional hazard model was used to estimate the hazard ratio (HR) of SAH associated with the treatment method and 95% confidence interval (CI). The model was adjusted for potential confounding factors, such as age, sex, health insurance type, surgical type (clipping or coiling), clinical status on admission (severe or mild), CCI, medical facility type (high-volume or low-volume hospitals), and stroke unit. The proportional hazards assumption was used to validate the Cox proportional hazards model. Data analysis was performed using SAS version 9.3 (SAS Institute Inc., Cary, NC, USA). A two-sided *P*-value of <0.05 was considered statistically significant.

## Results

### Baseline characteristics

Table 1 shows the baseline characteristics of the patients with SAH who were hospitalized during the follow-up period. In total, 2,634 patients underwent clipping and coiling within the follow-up period. Among them, 44.4% were 46–59 years old. The male to female ratio was 1:1.77. In total, 910 (34.5%) patients underwent clipping and 1,724 (65.5%) underwent coiling, and 395 (15.4%) had a severe clinical status on admission. A total of 2,543 (96.6%) patients had health insurance and 91 (3.4%) had medical aid. A total of 1,936 (73.5%) patients were admitted within 4.5 h after symptom onset, and 1,960 (74.4%) used an EMS to travel to the hospital. A total of 2,026 (76.9%) patients underwent CT or MRI within 1 h after admission to the emergency department; 1,407 (53.4%) and 1,227 (46.6%) patients were treated in tertiary and general hospitals, respectively, and 1,736 (65.9%) patients were treated in hospitals with stroke units. In total, 1,899 (72.1%) and 680 (25.8%) patients were discharged with good and poor functional outcomes, respectively. Functional outcome scales were not available for 55 (2.1%) patients, and functional outcome data were missing for 250 (9.5%) patients. The remaining function outcome at the discharge of patients with SAH is described in Supplementary Table 1.

**Table 1:** Baseline characteristics of patients with subarachnoid hemorrhage who underwent clipping and coiling.

**Variables**	**Total**	**Low volume**	**High volume**	***p*** **-value**
Total number of hospitals	136 (100%)	97 (71.3%)	39 (28.7%)	
Total number of patients	2,634 (100%)	1,090 (41.4%)	1,544 (58.6%)	
Age, years, mean ± SD	55.54 ± 12.77	55.10 ± 12.87	55.86 ± 12.69	<0.001^*^
18–45, *n* (%)	571 (21.68%)	253 (23.21%)	318 (20.60%)	0.134
46–59	1,169 (44.38%)	491 (45.05%)	678 (43.91%)	
60–69	489 (18.56%)	183 (16.79%)	306 (19.82%)	
≥70	405 (15.38%)	163 (14.95%)	242 (15.67%)	
Men, *n* (%)	952 (36.14%)	398 (36.51%)	554 (35.88%)	0.739
Women	1,682 (63.86%)	692 (63.49%)	990 (64.12%)	
**Health insurance type**, ***n*** **(%)**				
Health insurance	2,543 (96.55%)	1,051 (96.42%)	1,492 (96.63%)	0.771
Medical aid	91 (3.45%)	39 (3.58%)	52 (3.37%)	
NIHSS, mean ± SD	5.67 ± 10.09	5.64 ± 10.21	5.71 ± 9.99	
0–4, *n* (%)	348 (13.21%)	176 (16.15%)	172 (11.14%)	0.473
5–7	18 (0.68%)	12 (1.10%)	6 (0.39%)	
8–13	32 (1.21%)	13 (1.19%)	19 (1.23%)	
14–21	27 (1.03%)	15 (1.38%)	12 (0.78%)	
22–42	50 (1.90%)	24 (2.20%)	26 (1.68%)	
GCS, mean ± SD	13.00 ± 3.47	12.88 ± 3.67	13.08 ± 3.34	
0–8, *n* (%)	1,649 (62.60%)	642 (58.90%)	1,007 (65.22%)	0.035^*^
9–12	152 (5.77%)	49 (4.50%)	103 (6.67%)	
13–15	294 (11.16%)	131 (12.02%)	163 (10.56%)	
**Severity (NIHSS, GCS)**, ***n*** **(%)**				
Mild	2,175 (84.63%)	883 (83.15%)	1,292 (85.68%)	0.080
Severe	395 (15.37%)	179 (16.85%)	216 (14.32%)	
**Medical history**				
**Smoker**				
Current smoker, *n* (%)	402 (15.26%)	150 (13.76%)	252 (16.32%)	0.794
Ex-smoker	60 (2.28%)	25 (2.29%)	35 (2.27%)	
Non-smoker	1,158 (43.96%)	445 (40.83%)	713 (46.18%)	
**CCI score**, ***n*** **(%)**				
0	806 (30.60%)	288 (26.42%)	518 (33.55%)	<0.001^*^
1	844 (32.04%)	356 (32.66%)	488 (31.61%)	
2	478 (18.15%)	226 (20.73%)	252 (16.32%)	
≥3	506 (19.21%)	220 (20.18%)	286 (18.52%)	
**Arrival mode**, ***n*** **(%)**				
EMS	1,960 (74.41%)	813 (74.59%)	1,147 (74.29%)	0.884
No EMS	673 (25.55%)	277 (25.41%)	396 (25.65%)	
**Onset to door time**, ***n*** **(%)**				
≤ 4.5 h	1,936 (73.50%)	826 (75.78%)	1,110 (71.89%)	0.026^*^
>4.5 h	698 (26.50%)	264 (24.22%)	434 (28.11%)	
**Door to image time**, ***n*** **(%)**				
≤ 1 h	2,026 (76.92%)	840 (77.06%)	1,186 (76.81%)	0.880
>1 h	608 (23.08%)	250 (22.94%)	358 (23.19%)	
Tertiary hospital, *n* (%)	1,407 (53.42%)	291 (26.70%)	1,116 (72.28%)	<0.001^*^
General hospital	1,227 (46.58%)	799 (73.30%)	428 (27.72%)	
**Stroke unit**, ***n*** **(%)**				
Yes	1,736 (65.91%)	622 (57.06%)	1,114 (72.15%)	<0.001^*^
No	898 (34.09%)	468 (42.94%)	430 (27.85%)	
**Surgery type**, ***n*** **(%)**				
Clipping	910 (34.54%)	374 (34.31%)	536 (34.71%)	0.830
Coiling	1,724 (65.45%)	716 (65.69%)	1,008 (65.28%)	
**Functional outcome at discharge**, ***n*** **(%)**				
Good outcome	1,899 (72.10%)	743 (68.17%)	1,156 (74.87%)	0.002^*^
Poor outcome	680 (25.82%)	312 (28.62%)	368 (23.83%)	
**mRS**, ***n*** **(%)**				
0	481 (18.26%)	213 (19.54%)	268 (17.36%)	<0.001^*^
1	674 (25.59%)	230 (21.10%)	444 (28.76%)	
2	176 (6.68%)	61 (5.60%)	115 (7.45%)	
3	70 (2.66%)	20 (1.83%)	50 (3.24%)	
4	49 (1.86%)	14 (1.28%)	35 (2.27%)	
5	43 (1.63%)	14 (1.28%)	29 (1.88%)	
6	97 (3.68%)	49 (4.50%)	48 (3.11%)	
**GOS**, ***n*** **(%)**				
1	59 (2.24%)	27 (2.48%)	32 (2.07%)	0.278
2	12 (0.46%)	3 (0.28%)	9 (0.58%)	
3	19 (0.72%)	7 (0.64%)	12 (0.78%)	
4	51 (1.94%)	14 (1.28%)	37 (2.40%)	
5	463 (17.58%)	156 (14.31%)	307 (19.88%)	
**No. of deaths**, ***n*** **(%)**				
3-month (2013, 2014, 2016, 2018)	355 (13.48%)	168 (15.41%)	187 (12.11%)	0.015^*^
1-year (2013, 2014, 2016, 2018)	378 (14.35%)	178 (16.33%)	200 (12.95%)	0.015^*^
2-year (2013, 2014, 2016, 2018)	399 (15.15%)	189 (17.34%)	210 (13.60%)	0.008^*^
4-year (2013, 2014, 2016), N=1642	264 (16.08%)	123 (19.37%)	141 (14.00%)	0.004^*^

Values are presented as mean ± SD or n (%).

Mild severity, NIHSS ≤ 15, GCS ≥ 10.

Severe severity, NIHSS ≥ 16, GCS ≤ 9.

SD, standard deviation; NIHSS, National Institutes of Health Stroke Scale; GCS, Glasgow Coma Scale; EMS, emergency medical services; CCI, charlson comorbidity index; mRS, modified rankin scale; GOS, glasgow outcome scale; K-MBI, korean version of modified barthel index; MBI, modified barthel index; FIM, functional independence measure.

Good outcome: K-MBI (75–99), MBI (75–99), BI (75–99), mRS (0–2), FIM (90–126), GOS (5).

Patients with no record of functional outcome at discharge are excluded.

* Statistically significant p < 0.05.

### Mortality of patients with SAH

All patients (100%) were followed up for 2 years after the start of treatment. For full follow-up, patients diagnosed with SAH in 2018 were excluded due to a lack of 4-year mortality data (*n* = 992, 37.7%). The 3-month, 1, 2, and 4-year mortality rates of patients with SAH who underwent clipping and coiling were 13.5, 14.4, 15.2, and 16.1%, respectively.

**Figure 2:**
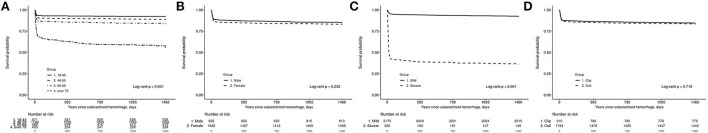
The Kaplan–Meier estimate for survival probability of 2,634 patients with subarachnoid hemorrhage (SAH) treated with clipping and coiling. **(A)** According to age, **(B)** according to sex, **(C)** according to severity, and **(D)** according to clipping and coiling group.

A KM analysis was performed to determine mortality according to hospital volume and treatment method in patients with SAH who were treated with clipping and coiling. Older patients had significantly higher mortality than younger patients (log-rank test, *p* < 0.001, Figure 2A). No statistically significant sex-specific differences were observed in the KM analysis (log-rank test, *p* = 0.230; Figure 2B). Patients with severe clinical status showed significantly higher mortality than those with mild clinical status (log-rank test, *p* < 0.001, Figure 2C). The KM analysis did not reveal any significant differences according to the types of surgery (clipping, coiling; log-rank test, *p* = 0.719, Figure 2D).

### Baseline characteristics by medical facility type

Of the 136 hospitals that performed clipping and coiling during the observation period, 39 (28.6%) were high-volume hospitals. In high-volume hospitals, 536 (34.7%) patients underwent clipping and 1,008 (65.3%) underwent coiling. In low-volume hospitals, 374 (34.3%) patients underwent clipping and 716 (65.7%) underwent coiling. Clipping and coiling frequencies did not significantly differ between high- and low-volume hospitals (*p* > 0.05). The distribution of age, sex ratio, clinical status, and health insurance type did not differ between high-volume and low-volume hospitals (*p* > 0.05). Although the groups did not differ in terms of a medical history of smoking, compared to low-volume hospitals, high-volume hospitals had a significantly higher proportion of patients with low CCI (CCI 0) (26.42 vs. 33.55%, *p* < 0.001) and stroke units (57.1 vs. 72.2%, *p* < 0.001) (Table 1).

### Distribution of patients according to treatment method

Supplementary Figure 1 shows the distribution of patients according to the treatment method in 2013, 2014, 2016, and 2018. The number of patients who underwent clipping was 197 (47.4%) in 2013, 122 (41.5%) in 2014, 320 (34.3%) in 2016, and 271 (27.3%) in 2018. The number of patients who underwent coiling was 219 (52.6%) in 2013, 172 (58.5%) in 2014, 612 (65.7%) in 2016, and 721 (72.7%) in 2018. The proportion of patients who underwent coiling is increasing annually.

Supplementary Figure 2 shows the distribution of overall patients according to the treatment method in high-volume and low-volume hospitals. In high-volume hospital, the number of patients who underwent clipping were 118 (48.2%) in 2013, 73 (38.4%) in 2014, 198 (34.6%) in 2016, and 147 (27.4%) in 2018. The number of patients who underwent coiling were 127 (51.8%) in 2013, 117 (61.6%) in 2014, 374 (65.4%) in 2016, and 390 (72.6%) in 2018. In low-volume hospitals, the number of patients who underwent clipping were 79 (46.2%) in 2013, 49 (47.1%) in 2014, 122 (33.9%) in 2016, and 124 (27.3%) in 2018. The number of patients who underwent coiling were 92 (53.8%) in 2013, 55 (52.9%) in 2014, 238 (66.1%) in 2016, and 331 (72.7%) in 2018. The proportion of coiling was higher in both high-volume and low-volume hospitals.

### Mortality according to medical facility type

The 3-month, 1, 2, and 4-year mortality rates in high-volume hospitals were 12.1, 13.0, 13, and 14.0%, respectively, while they were 15.4, 16.3, 17.3, and 19.4%, respectively, in low-volume hospitals. Patients with SAH in high-volume hospitals had significantly lower short-term (3-month, 1-year) and long-term mortality (2, 4-year) (*p* < 0.05).

**Figure 3:**
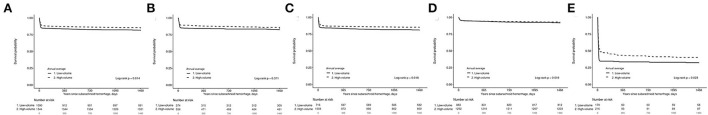
Kaplan–Meier estimate for survival probability of 2,634 patients with subarachnoid hemorrhage (SAH) treated with clipping and coiling. **(A)** According to hospital type, **(B)** 910 patients treated with clipping according to hospital type, **(C)** 1,724 patients treated with coiling according to hospital type, **(D)** Mild patients according to hospital type, and **(E)** Severe patients according to hospital type.

In the KM survival analysis, high-volume hospitals showed significantly higher survival rates than low-volume hospitals (log-rank test, *p* = 0.014, Figure 3A). The survival rates of patients treated with clipping did not differ significantly between high-volume and low-volume hospitals (*p* = 0.371, Figure 3B); however, KM survival analysis showed that patients treated with coiling after SAH in high-volume hospitals had significantly higher survival rates than those in low-volume hospitals (*p* = 0.018, Figure 3C). For patients with mild clinical status, KM analysis did not show any difference in survival rate between high-volume and low-volume hospitals (*p* = 0.519, Figure 3D). However, for severe clinical status, high-volume hospitals had a significantly higher survival rate than low-volume hospitals (*p* = 0.023, Figure 3E).

### Multivariate logistic analysis of functional outcome at discharge

The results of the multivariate logistic analysis predicting poor functional outcomes at discharge are displayed in Supplementary Table 2. After all covariates, including the severity of SAH, were adjusted, treatment in high-volume hospitals was associated with a lower odds ratio (OR) of poor outcome at discharge (OR, 0.77; 95% CI, 0.62–0.95, *p* = 0.017) compared with low-volume hospitals. Compared with clipping, coiling was not associated with a poorer outcome at discharge (OR, 0.99; 95% CI, 0.79–1.23, *p* = 0.896). Severe clinical status was associated with higher odds of poor outcomes at discharge (OR, 14.01; 95% CI, 10.58–18.56, *p* < 0.001) compared with mild clinical status. An age of more than 70 years was associated with higher odds of poor outcomes at discharge (OR, 4.45; 95% CI, 3.07–6.44, *p* < 0.001) than an age between 18 and 45 years. Arrival at the hospital without EMS was associated with lower odds of poor outcomes at discharge (OR, 0.45; 95% CI, 0.34–0.60, *p* < 0.001) compared with arrival *via* EMS. Patients with CCIs of ≥3 had higher odds of poor outcomes at discharge (OR, 2.03; 95% CI, 1.49–2.75; *p* < 0.001) than those with CCI scores of 0.

### Cox analysis of death of patients with SAH who underwent clipping and coiling

Supplementary Table 3 shows the results of Cox analysis of death in patients with SAH who underwent clipping and coiling during short- and long-term follow-up. High-volume hospitals had significantly lower 3-month mortality rates than low-volume hospitals (HR, 95% CI, 0.75, 0.62–0.92, *p* = 0.0045). High-volume hospitals had lower 1-, 2-, and 4-year mortality rates than low-volume hospitals, but the difference was not significant (*p* > 0.05). Mortality did not differ significantly between clipping and coiling for patients with SAH. Patients aged >70 years had significantly higher 3-month mortality than those aged 18–45 years (HR, 95% CI, 1.91, 1.32–2.75, *p* = 0.0006). Patients aged >70 had higher 1-, 2-, and 4-year mortality rates, but the difference was not statistically significant (*p* > 0.05). Mortality did not differ according to sex and health insurance type (*p* > 0.05). Patients who arrived at the hospital without EMS had lower 3-month mortality than those who arrived at the hospital with EMS (HR, 95% CI, 0.71, 0.50–1.00). CCI did not differ significantly at the 3-month follow-up; however, patients with CCI > 3 had significantly lower 1-, 2-, and 4-year mortality than those with CCI 0 (1-year HR, 95% CI, 0.54, 0.41–0.73; 2-year 0.62, 0.47–0.83; 4-year 0.60, 0.42–0.85).

### Cox analysis of death according to patient severity

Table 2 shows the Cox analysis of death in SAH patients with mild and severe clinical statuses who underwent clipping and coiling during short- and long-term follow-ups. In patients with mild clinical status, high-volume hospitals had significantly lower 3-month mortality (HR, 95% CI, 0.67, 0.49–0.92, *p* = 0.0133), but high-volume hospitals did not have significant differences in 1-, 2-, and 4-year mortality compared to low-volume hospitals (*p* > 0.05). Clipping and coiling did not show significant difference in mortality in patients with a mild clinical status (*p* > 0.05). Patients aged >70 years had significantly higher 3-month and 1-year mortality rates than those aged 18–45 years (3-month HR, 95% CI, 3.71, 2.00–6.88, *p* < 0.0001; 1-year 1.96, 1.05–3.64; *p* = 0.0348). Neither did the 2-year and 4-year mortality rates differ significantly (*p* > 0.05) nor did sex or health insurance type in the mild clinical status group (*p* > 0.05). In patients with mild clinical status, those who did not visit the hospital *via* EMS had significantly lower 3-month and 1-year mortality rates than those who received EMS (3-month HR, 95% CI, 0.49, 0.31–0.77, *p* = 0.0020; 1-year 0.56, 0.36–0.87; *p* = 0.0102). The 2- and 4-year mortality rates of patients who came to the hospital *via* EMS were lower, but the difference was not statistically significant (*p* > 0.05). Patients with mild clinical status and CCI >3 had significantly lower 1- and 2-year mortality rates than those with CCI 0 (1-year HR, 95% CI, 0.54, 0.34–0.86, *p* = 0.0095; 2-year 0.63, 0.40–0.99; *p* = 0.0469).

**Table 2:** Cox analysis of death according to severity in patients with subarachnoid hemorrhage for 4 years.

	**3 month**		**1 year**		**2 year**		**4 year**	
	**Mild**	**Severe**	**Mild**	**Severe**	**Mild**	**Severe**	**Mild**	**Severe**
	**HR(95% CI)**	**HR(95% CI)**	**HR(95% CI)**	**HR(95% CI)**	**HR(95% CI)**	**HR(95% CI)**	**HR(95% CI)**	**HR(95% CI)**
**Medical facility type**								
Low-volume hospitals	1.0	1.0	1.0	1.0	1.0	1.0	1.0	1.0
High-volume hospitals	0.67 (0.49–0.92)^*^	0.79 (0.61–1.03)	0.92 (0.67–1.26)	0.76 (0.58–0.99)^*^	0.99 (0.01–0.72)	0.72 (0.55–0.94)^*^	0.94 (0.31–0.84)	0.84 (0.60–1.18)
**Surgery type**								
Clipping	1.0	1.0	1.0	1.0	1.0	1.0	1.0	1.0
Coiling	0.85 (0.62–1.17)	0.97 (0.74–1.27)	0.77 (0.56–1.05)	1.13 (0.86–1.49)	0.89 (0.65–1.21)	1.07 (0.82–1.40)	0.83 (0.57–1.20)	1.22 (0.87–1.71)
**Age, years**								
18–45	1.0	1.0	1.0	1.0	1.0	1.0	1.0	1.0
46–59	1.72 (0.97–3.06)	1.15 (0.75–1.78)	1.18 (0.67–2.10)	1.17 (0.76–1.81)	1.44 (0.81–2.56)	1.17 (0.76–1.80)	1.12 (0.55–2.24)	0.86 (0.50–1.48)
60–69	2.23 (1.18–4.24)^*^	1.26 (0.77–2.06)	1.96 (1.04–3.69)^*^	0.84 (0.51–1.38)	1.79 (0.96–3.31)	0.72 (0.44–1.18)	1.83 (0.87–3.83)	0.88 (0.48–1.62)
≥70	3.71 (2.00–6.88)^***^	1.34 (0.84–2.13)	1.96 (1.05–3.64)^*^	0.90 (0.56–1.45)	1.54 (0.86–2.76)	0.85 (0.53–1.36)	1.87 (0.92–3.81)	0.79 (0.43–1.42)
Men	1.0	1.0	1.0	1.0	1.0	1.0	1.0	1.0
Women	0.70 (0.48–1.03)	0.92 (0.69–1.24)	0.88 (0.61–1.29)	1.19 (0.89–1.59)	1.14 (0.78–1.78)	1.23 (0.92–1.64)	0.83 (0.52–1.31)	1.38 (0.96–2.00)
**Health insurance type**								
Health insurance	1.0	1.0	1.0	1.0	1.0	1.0	1.0	1.0
Medical aid	1.20 (0.64–2.23)	0.94 (0.55–1.59)	1.38 (0.74–2.57)	1.28 (0.76–2.17)	0.90 (0.49–1.67)	1.31 (0.77–2.22)	1.42 (0.67–3.00)	1.32 (0.67–2.58)
**Arrival mode**								
EMS	1.0	1.0	1.0	1.0	1.0	1.0	1.0	1.0
No EMS	0.49 (0.31–0.77)^**^	1.61 (0.97–2.67)	0.56 (0.36–0.87)^*^	1.89 (1.13–3.15)^*^	0.67 (0.43–1.04)	1.14 (0.67–1.95)	0.76 (0.44–1.30)	1.38 (0.80–2.39)
**Medical history**								
**CCI score**								
0	1.0	1.0	1.0	1.0	1.0	1.0	1.0	1.0
1	0.84 (0.54–1.29)	0.91 (0.64–1.31)	0.83 (0.54–1.30)	0.82 (0.57–1.19)	1.04 (0.66–1.63)	0.98 (0.68–1.41)	0.80 (0.47–1.38)	1.06 (0.67–1.68)
2	1.15 (0.72–1.82)	0.81 (0.55–1.18)	1.22 (0.77–1.94)	0.69 (0.47–1.01)	0.87 (0.54–1.42)	0.79 (0.53–1.16)	1.09 (0.62–1.92)	0.65 (0.40–1.07)
≥3	1.06 (0.69–1.64)	0.71 (0.49–1.03)	0.54 (0.34–0.86)^**^	0.53 (0.36–0.78)^***^	0.63 (0.40–0.99)^*^	0.62 (0.43–0.91)^*^	0.76 (0.45–1.30)	0.58 (0.37–0.93)^*^

Values are presented as HR (95% CI), HR, hazard ratio; 95% CI, 95% confidence intervals.

EMS, emergency medical services; CCI, Charlson comorbidity index.

* Statistically significant p < 0.05.

** Statistically significant p < 0.01.

*** Statistically significant p < 0.001.

In terms of severe clinical status, high-volume hospitals had lower 3-month and 4-year mortality, but the difference was not statistically significant (*p* > 0.05). However, high-volume hospitals had significantly lower 1-year and 2-year mortality rates (1-year HR, 95% CI, 0.76, 0.58–0.99, *p* = 0.0425; 2-year 0.72, 0.55–0.94; *p* = 0.0144). Clipping and coiling did not result in significant differences in mortality in patients with severe clinical statuses (*p* > 0.05). Age, sex, and health insurance type did not differ significantly in patients with severe clinical status (*p* > 0.05). Patients who did not visit the hospital *via* EMS had higher 3-month, 2-year, and 4-year mortality rates, but the difference was not statistically significant (*p* > 0.05). However, patients who did not visit the hospital *via* EMS had a significantly higher 1-year mortality than those who did (HR, 95% CI, 1.89, 1.13–3.15, *p* = 0.0147). Patients with CCI > 3 had significantly lower 1-, 2-, and 4-year mortality rates than patients with CCI 0 (1-year HR, 95% CI, 0.53, 0.36–0.78, *p* = 0.0010; 2-year 0.62, 0.43–0.91, *p* = 0.0135; 4-year 0.58, 0.37–0.93; *p* = 0.0228).

### Hospital costs of patients with SAH

We analyzed the hospital cost of 2,634 patients with SAH. Table 3 shows the hospital costs by volume and surgical treatment. The mean total cost was $23,408 ± 13,179 (mean ± SD; range $6,788–$205,659). One-thousand-and-ninety patients were enrolled in low-volume hospitals, and the total cost was $22,510 ± 11,195 (mean ± SD; range, $6,985–$156,817), whereas 1,544 patients were enrolled in high-volume hospitals, and the total cost was $24,042.01 ± 14,387.03 (mean ± SD; range $6,788–$205,659). Due to the cost analysis according to surgery, the cost in high-volume hospitals was significantly higher than that in low-volume hospitals (*p* < 0.05). Coiling was significantly more expensive than clipping ($24,133 ± 13,566 vs. $22,033 ± 12,304, *p* < 0.05). Clipping was significantly more expensive in high-volume hospitals than in low-volume hospitals ($22,715 ± 14,282 vs. $21,056 ± 8,645, *p* = 0.0297). Coiling was also more expensive in high-volume hospitals than in low-volume hospitals ($24,747 ± 14,399 vs. $23,269 ± 12,257, *p* = 0.0220).

Table 3Hospital costs by hospital volume and surgical treatment of 2,634 patients.

**Table:** 

	***N***	**Average**	**Standard deviation**	**Min**	**Max**	***p*** **-value**
Total	2,634	$23,408.06	$13,179.94	$6,788.39	$205,659.05	0.002
Low-volume hospital	1,090	$22,510.06	$11,195.74	$6,985.27	$156,817.65	
High-volume hospital	1,544	$24,042.01	$14,387.03	$6,788.39	$205,659.05

**Table:** 

	**Total**	**Low-volume hospital**	**High-volume hospital**	***p*** **-value**
Total	$23,408.06 ± 13,179.94	$22,510.06 ± 11,195.74	$24,042.01 ± 14,387.03	0.002
Clip	$22,033.69 ± 12,304.22	$21,056.28 ± 8,645.06	$22,715.69 ± 14,282.43	0.030
Coil	$24,133.50 ± 13,566.91	$23,269.43 ± 12,257.16	$24,747.27 ± 14,399.70	0.022

Cost is calculated on the Korean Won (KRW 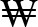) then converted to the United States dollar (USD $) using an exchange rate of USD $1 = KRW 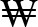
808 according to purchasing power parties of 2022 on the OECD.

## Discussion

A previous study that analyzed patients with SAH in Korea in 2013 and 2014 revealed 1- and 5-year mortality rates of 21.4 and 24.3%, respectively (8). This study analyzed patients in 2013, 2014, 2016, and 2018, including data after 2015, and revealed 1- and 4-year mortality rates of 14.4 and 16.1%, which were lower than those observed previously. This change shows a similar trend to the 33–20% reduction in mortality rate reported among American patients with SAH (16, 17). Our study includes data from after 2015, and the mortality rate was lower than that reported in previous studies that used data from before 2015. As endovascular techniques and neurocritical care in SAH surgery have advanced over time, the mortality rate of patients with SAH has decreased.

In this study, high-volume hospitals had high proportions of stroke units, representing good patient care environments (72.2 vs. 57.1%, *p* < 0.001). Similar to a previous study showing that high-volume hospitals led patients to good outcomes, reports also show that larger hospitals are associated with better outcomes in SAH patients requiring complex surgery (8, 9, 18). The general principle of high-quality care and the outcomes at high-volume centers arise from the improvement in the skills and experience of surgeons and other assistants across numerous procedures. Postoperative care, such as triple-H therapy, cardiopulmonary care, and hourly checkups of neurological status, can easily be performed in high-quality intensive care or stroke units with well-trained residents and nurses. Based on the clinical status, improved clinical outcomes in high-volume centers may reflect appropriate planning and treatment algorithms for treating ruptured aneurysms after SAH. Similar to a previous study, patients with SAH had better outcomes in high-volume hospitals.

In our study, differences in clipping and coiling did not result in a significant difference in mortality. Treatment for ruptured aneurysms is divided into clipping and coiling, which are difficult procedures and involve a steep learning curve that involves the temporary removal of a bone flap from the skull and ligation of the ruptured portion of the aneurysm. The International Subarachnoid Aneurysm Trial (ISAT) demonstrated the superiority of coiling over clipping with lower 1-year mortality and epilepsy rates (19, 20). More randomized controlled trials (RCTs) and prospective and retrospective studies have been published since the ISAT, some of which have reported results that differ from those of the ISAT (21). O'Kelly et al. retrospectively analyzed a cohort of adult patients with aneurysmal SAH who received treatment for aneurysms in Ontario between 1995 and 2004. In addition, contrary to the general findings of previous studies, it was found that patients who underwent clipping had a significantly lower mortality rate than those who underwent coiling (22). In the barrow ruptured aneurysm trial (BRAT), clipping and coiling did not differ significantly in terms of clinical outcomes (mRS outcomes and deaths). The superiority of both treatment outcomes is still controversial for each of these methods. In our study, the short- and long-term mortality of patients with SAH who underwent clipping and coiling did not differ significantly. They may not represent a real clinical environment as both ISAT and BRAT are RCTs. However, because our study was a large observational study, it represents an actual clinical environment. In our study, there was no significant difference in mortality rates after clipping and coiling.

Although there was no difference in mortality rates between clipping and coiling in our study, as a result of examining the rates of clipping and coiling for each year, the number of patients who received coiling is steadily increasing. After ISAT, the proportion of coiling in Korea increased and the proportion of coiling in both high-volume and low-volume increased (Supplementary Table 3).

The results showed that the 3-month mortality rate in the group aged >70 years was significantly higher than that in those aged 18–45 years. Previous studies that included only data from before 2015 showed that the 3-month, 1, and 2-year mortality rates were significantly higher in the older patient group. However, in the present study, only the 3-month mortality rates were significantly higher in the older patient group. Unlike previous results showing that the >70 years age group has higher mortality rates and more expensive hospital fees, the mortality rate between the old and young age groups did not differ significantly (23). The development of SAH treatment has changed the mortality rate with age.

CCI is unsuitable for measuring a patient's condition because, by definition, CCI is associated with specific comorbidities and not with neurological conditions (24, 25). In SAH, the initial clinical status and intracranial pressure are more important than CCI.

Hospital costs differed significantly between high- and low-volume hospitals. High-volume hospitals had significantly higher costs than low-volume hospitals for all patients, including in terms of classification according to clipping and coiling. In a previous cost-effectiveness analysis of SAH patients, the cost of clipping and coiling in the USA in 2013 was $93,597 and $87,441, respectively. In Korea in 2015, clipping and coiling costs were $17,221 ± 7,732 and $20,671 ± 9,704, respectively. The treatment cost for SAH in Korea was lower than that in the USA. The treatment cost in Korea was 82% and 77% lower for clipping and coiling, respectively. In research on the paradigm shift in ruptured aneurysms in the USA, clipping was used to treat most ruptured aneurysms, and the proportion of aneurysms decreased over time. Conversely, the proportion of coiling is increasing gradually (26). Korea also developed its coiling technology according to this paradigm, and the cost of coiling in Korea is higher than that of clipping, as observed previously (27). In Korea, the national health insurance system is in operation, and HIRA adjusts the medical cost. Using an exchange rate of USD $1 = KRW 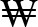
808 based on the PPP of 2022, the price of one clip is $185 and the price of one coil is $742, and these prices differ from those of other countries. The difference in medical costs, including staff costs, resulted in the reversal of the price differences between clipping and coiling. Coiling costs are higher than those of clipping in Korea. According to the cost analysis, the difference between the different insurance systems in each country was the cost difference.

Although this study analyzes nationwide data, it had several limitations. First, the data did not include brain images; therefore, we could not assess stroke severity. Therefore, we used the NIHSS and GCS scores from prospectively collected data to adjust for stroke severity (28). The International Cooperative Study on the Timing of Aneurysm Surgery has shown that consciousness level is important in predicting death and disability. The GCS is globally recognized and has been used previously to assess the level of consciousness in patients with SAH who initially underwent aneurysmal surgery (29–31). Therefore, we believe that the ASAR should be examined to determine the neurological status of patients with SAH on initial admission using GCS. The GCS score could be a representative measure to assess the outcomes of patients with SAH. Second, high-volume hospitals were represented according to the number of clipping and coiling cases per year in each hospital. The best method for classifying high-volume and low-volume hospitals remains controversial; therefore, the criteria were set by referring to previous studies that compared mortality rates according to hospital volume (8, 9). High-volume hospitals in our research were defined as having a high number of cases, and the results showed an association between high-volume hospitals and lower mortality in patients with severe clinical status (32). Third, since this study included data up to December 2018, a full 4-year follow-up could not be performed. Therefore, some patient data were omitted when the 4-year mortality rate was measured. Because only a subset of patients was used to measure the 4-year mortality, the 4-year mortality rate may not be significant. Therefore, a follow-up study using all follow-up data is necessary. Fourth, due to the difference in the number of patients between clipping and coiling, coiling showed a difference in mortality between hospital volumes, but clipping did not show any difference in mortality according to the difference in hospital volume. A retrospective study with a larger number of patients is needed, and both clipping and coiling are expected to show differences in mortality according to hospital size differences.

## Conclusion

Patients with SAH who underwent clipping and coiling, especially those with severe clinical status, had lower short- and long-term mortality rates in high-volume hospitals than in low-volume hospitals. The Cox analysis showed that short-term mortality in high-volume hospitals was lower than that in low-volume hospitals. Based on a nationwide database from our results, there was no difference in mortality between clipping and coiling in our study, regardless of whether patients were treated with high or low volume. Based on the analysis method adjusted for other variables, it can be inferred that SAH should be treated by an experienced neurosurgeon with high-quality facilities.

## Data availability statement

The data analyzed in this study was obtained from the Acute Stroke Assessment Registry (ASAR), the following licenses/restrictions apply: The data is encrypted and stored by the Health Insurance Review and Assessment Service (HIRA), and can only be accessed and analyzed within a specified period *via* a data application. Requests to access these datasets should be directed to HIRA, https://opendata.hira.or.kr/home.do.

## Ethics statement

The studies involving human participants were reviewed and approved by Research Ethics Committee of Soonchunhyang University Hospital. Written informed consent for participation was not required for this study in accordance with the national legislation and the institutional requirements.

## Author contributions

ML and JO conceived the study. S-WP, JL, ML, and JO researched the literature. All authors co-wrote and revised the article for intellectual content as well as provided approval for article submission and reviewed and approved the manuscript.

## Funding

This research was funded by Soonchunhyang University Fund. This research was supported by the Bio & Medical Technology Development Program of the National Research Foundation funded by the Government of the Republic of Korea (NRF-2019M3E5D1A02069061) and by the Korea Medical Device Development Fund grant funded by the Korean Government (202015X17). The funding sources had no role in the design and conduct of the study; collection, management, analysis, and interpretation of the data; preparation, review, or approval of the manuscript; and the decision to submit the manuscript for publication.

## Conflict of interest

The authors declare that the research was conducted in the absence of any commercial or financial relationships that could be construed as a potential conflict of interest.

## Publisher's note

All claims expressed in this article are solely those of the authors and do not necessarily represent those of their affiliated organizations, or those of the publisher, the editors and the reviewers. Any product that may be evaluated in this article, or claim that may be made by its manufacturer, is not guaranteed or endorsed by the publisher.

## References

[1] Kim JY Kang K Kang J Koo J Kim DH Kim BJ. Executive summary of stroke statistics in Korea 2018: a report from the epidemiology research council of the Korean stroke society. J Stroke. (2019) 21:42–59. 10.5853/jos.2018.03125 30558400 PMC6372894

[2] Pobereskin LH. Incidence and outcome of subarachnoid haemorrhage: a retrospective population based study. J Neurol Neurosurg Psychiatry. (2001) 70:340–3. 10.1136/jnnp.70.3.340 11181855 PMC1737269

[3] Chan V O'Kelly C. Response by Chan and O'Kelly to letter regarding article. Declining admission and mortality rates for subarachnoid hemorrhage in Canada between 2004 and 2015. Stroke. (2019) 50:e133. 10.1161/STROKEAHA.119.025114 30909838

[4] Suarez JI Zaidat OO Suri MF Feen ES Lynch G Hickman J. Length of stay and mortality in neurocritically ill patients: impact of a specialized neurocritical care team. Crit Care Med. (2004) 32:2311–7. 10.1097/01.CCM.0000146132.29042.4C 15640647

[5] Bogason ET Anderson B Brandmeir NJ Church EW Cooke J Davies GM. The epidemiology of admissions of nontraumatic subarachnoid hemorrhage in the United States. Neurosurgery. (2014) 74:227–9. 10.1227/NEU.0000000000000240 24435139

[6] Spetzler RF McDougall CG Zabramski JM Albuquerque FC Hills NK Russin JJ. The barrow ruptured aneurysm trial: 6-year results. J Neurosurg. (2015) 123:609–17. 10.3171/2014.9.JNS141749 26115467

[7] Spetzler RF McDougall CG Zabramski JM Albuquerque FC Hills NK Nakaji P. Ten-year analysis of saccular aneurysms in the barrow ruptured aneurysm trial. J Neurosurg. (2019) 132:771–6. 10.3171/2018.8.JNS181846 30849758

[8] Lee JY Heo NH Lee MR Ahn JM Oh HJ Shim JJ. Short and long-term outcomes of subarachnoid hemorrhage treatment according to hospital volume in Korea: a nationwide multicenter registry. J Korean Med Sci. (2021) 36:e146. 10.3346/jkms.2021.36.e146 34100560 PMC8185126

[9] Rush B Romano K Ashkanani M McDermid RC Celi LA. Impact of hospital case-volume on subarachnoid hemorrhage outcomes: a nationwide analysis adjusting for hemorrhage severity. J Crit Care. (2017) 37:240–3. 10.1016/j.jcrc.2016.09.009 27663296 PMC5679218

[10] Starke RM Komotar RJ Kim GH Kellner CP Otten ML Hahn DK. Evaluation of a revised Glasgow Coma Score scale in predicting long-term outcome of poor grade aneurysmal subarachnoid hemorrhage patients. J Clin Neurosci. (2009) 16:894–9. 10.1016/j.jocn.2008.10.010 19375327

[11] Hacke W Albers G Al-Rawi Y Bogousslavsky J Davalos A Eliasziw M. The desmoteplase in Acute ischemic Stroke Trial (DIAS): a phase II MRI-based 9-hour window acute stroke thrombolysis trial with intravenous desmoteplase. Stroke. (2005) 36:66–73. 10.1161/01.STR.0000149938.08731.2c 15569863

[12] Luk JK Cheung RT Ho SL Li L. Does age predict outcome in stroke rehabilitation? A study of 878 Chinese subjects. Cerebrovasc Dis. (2006) 21:229–34. 10.1159/000091219 16446535

[13] Sobrino T Hurtado O Moro MA Rodríguez-Yáñez M Castellanos M Brea D. The increase of circulating endothelial progenitor cells after acute ischemic stroke is associated with good outcome. Stroke. (2007) 38:2759–64. 10.1161/STROKEAHA.107.484386 17761925

[14] Mailles A De Broucker T Costanzo P Martinez-Almoyna L Vaillant V Stahl JP. Long-term outcome of patients presenting with acute infectious encephalitis of various causes in France. Clin Infect Dis. (2012) 54:1455–64. 10.1093/cid/cis226 22460967

[15] Yoneda Y Okuda S Hamada R Toyota A Gotoh J Watanabe M. Hospital cost of ischemic stroke and intracerebral hemorrhage in Japanese stroke centers. Health Policy. (2005) 73:202–11. 10.1016/j.healthpol.2004.11.016 15978963

[16] Rincon F Rossenwasser RH Dumont A. The epidemiology of admissions of nontraumatic subarachnoid hemorrhage in the United States. Neurosurgery. (2013) 73:217–22. 10.1227/01.neu.0000430290.93304.33 23615089

[17] Neifert SN Martini ML Hardigan T Ladner TR MacDonald RL Oermann EK. Trends in incidence and mortality by hospital teaching status and location in aneurysmal subarachnoid hemorrhage. World Neurosurg. (2020) 142:e253–9. 10.1016/j.wneu.2020.06.180 32599190

[18] Birkmeyer JD Siewers AE Finlayson EV Stukel TA Lucas FL Batista I. Hospital volume and surgical mortality in the United States. N Engl J Med. (2002) 346:1128–37. 10.1056/NEJMsa012337 11948273

[19] Molyneux A Kerr R Stratton I Sandercock P Clarke M Shrimpton J. International Subarachnoid Aneurysm Trial (ISAT) of neurosurgical clipping versus endovascular coiling in 2143 patients with ruptured intracranial aneurysms: a randomised trial. Lancet. (2002) 360:1267–74. 10.1016/S0140-6736(02)11314-6 12414200

[20] Alshekhlee A Mehta S Edgell RC Vora N Feen E Mohammadi A. Hospital mortality and complications of electively clipped or coiled unruptured intracranial aneurysm. Stroke. (2010) 41:1471–6. 10.1161/STROKEAHA.110.580647 20522817

[21] Li H Pan R Wang H Rong X Yin Z Milgrom DP. Clipping versus coiling for ruptured intracranial aneurysms: a systematic review and meta-analysis. Stroke. (2013) 44:29–37. 10.1161/STROKEAHA.112.663559 23238862

[22] O'Kelly CJ Kulkarni AV Austin PC Wallace MC Urbach D. The impact of therapeutic modality on outcomes following repair of ruptured intracranial aneurysms: an administrative data analysis. Clin Article Clin Article J Neurosurg. (2010) 113:795–801. 10.3171/2009.9.JNS081645 19852537

[23] Lanzino G Kassell NF Germanson TP Kongable GL Truskowski LL Torner JC. Age and outcome after aneurysmal subarachnoid hemorrhage: why do older patients fare worse? J Neurosurg. (1996) 85:410–8. 10.3171/jns.1996.85.3.0410 8751625

[24] Washington CW Derdeyn CP Dacey RG Dhar R Zipfel GJ. Analysis of subarachnoid hemorrhage using the nationwide inpatient sample: the NIS-SAH Severity score and outcome measure. J Neurosurg. (2014) 121:482–9. 10.3171/2014.4.JNS131100 24949676

[25] Boogaarts HD Conde MP Janssen E van Nuenen WF de Vries J Donders R. The value of the Charlson Co-morbidity Index in aneurysmal subarachnoid haemorrhage. Acta Neurochir (Wien). (2014) 156:1663–7. 10.1007/s00701-014-2160-3 24973200

[26] Lin N Cahill KS Frerichs KU Friedlander RM Claus EB. Treatment of ruptured and unruptured cerebral aneurysms in the USA: a paradigm shift. J Neurointerv Surg. (2018) 10:i69–76. 10.1136/jnis.2011.004978.rep 30037962

[27] Zhang X Li L Hong B Xu Y Liu Y Huang Q. A systematic review and meta-analysis on economic comparison between endovascular coiling versus neurosurgical clipping for ruptured intracranial aneurysms. World Neurosurg. (2018) 113:269–75. 10.1016/j.wneu.2018.02.078 29476995

[28] Charlson ME Pompei P Ales KL MacKenzie CR. A new method of classifying prognostic comorbidity in longitudinal studies: development and validation. J Chronic Dis. (1987) 40:373–83. 10.1016/0021-9681(87)90171-8 3558716

[29] Gotoh O Tamura A Yasui N Suzuki A Hadeishi H Sano K. Glasgow coma scale in the prediction of outcome after early aneurysm surgery. Neurosurgery. (1996) 39:19–24. 10.1097/00006123-199607000-00005 8805136

[30] Kassell NF Torner JC Haley EC Jane JA Adams HP Kongable GL. The international cooperative study on the timing of aneurysm surgery. Part 1: overall management results. J Neurosurg. (1990) 73:18–36. 10.3171/jns.1990.73.1.0018 2191090

[31] Kassell NF Torner JC Jane JA Haley EC Jr Adams HP. The international cooperative study on the timing of aneurysm surgery. Part 2: surgical results. J Neurosurg. (1990) 73:37–47. 10.3171/jns.1990.73.1.0037 2191091

[32] Pandey AS Gemmete JJ Wilson TJ Chaudhary N Thompson BG Morgenstern LB. High subarachnoid hemorrhage patient volume associated with lower mortality and better outcomes. Neurosurgery. (2015) 77:462–70. 10.1227/NEU.0000000000000850 26110818 PMC4869982

