# Supplementary material for: Short- and long-term mortality of subarachnoid hemorrhage according to hospital volume and severity using a nationwide multicenter registry study
Source: Front Neurol. 2022 Aug 5;13:952794. doi: 10.3389/fneur.2022.952794 (PMC9389169; doi:10.3389/fneur.2022.952794)
Supplement: Supplementary file 1 [file Table_1.DOCX]

| Variables | Total | Low-volume | High-volume |
| --- | --- | --- | --- |
| Total number of patients | 2,634(100%) | 1,090(41.4%) | 1,544(58.6%) |
| functional outcome at discharge, n(%) |  |  |  |
| Good outcome | 1,899(72.10%) | 743(68.17%) | 1,156(74.87%) |
| Bad outcome | 680(25.82%) | 312(28.62%) | 368(23.83%) |
| K-MBI, n(%) |  |  |  |
| Good outcome | 47(1.78%) | 28(2.57%) | 19(1.23%) |
| Bad outcome | 18(0.68%) | 6(0.55%) | 12(0.78%) |
| MBI, n(%) |  |  |  |
| Good outcome | 48(1.82%) | 45(4.13%) | 3(0.19%) |
| Bad outcome | 10(0.38%) | 8(0.73%) | 2(0.13%) |
| BI, n(%) |  |  |  |
| Good outcome | 10(0.38%) | 10(0.92%) | 0(0.00%) |
| Bad outcome | 1(0.04%) | 1(0.09%) | 0(0.00%) |
| FIM, n(%) |  |  |  |
| Good outcome | 0(0.00%) | 0(0.00%) | 0(0.00%) |
| Bad outcome | 1(0.04%) | 0(0.00%) | 1(0.06%) |

Supplementary Table 1. Details of the functional outcome at discharge in patients with subarachnoid hemorrhage.

K-MBI=Korean Version of Modified Barthel Index, MBI=Modified Barthel Index, BI=Barthel Index, FIM=Functional Independence Measure

Good Outcome: K-MBI (75–99), MBI (75–99), BI (75–99), mRS (0–2), FIM (90–126), GOS (5)
