# Supplementary material for: Short- and long-term mortality of subarachnoid hemorrhage according to hospital volume and severity using a nationwide multicenter registry study
Source: Front Neurol. 2022 Aug 5;13:952794. doi: 10.3389/fneur.2022.952794 (PMC9389169; doi:10.3389/fneur.2022.952794)
Supplement: Supplementary file 2 [file Table_2.DOCX]

Supplementary Table 2. Results of multivariate logistic regression analysis of poor functional outcome at discharge.

|  | Poor outcome at discharge | |
| --- | --- | --- |
|  | OR(95% CI) | |
|  | OR(95% CI) | p-value |
| Medical facility type |  |  |
| Low-volume hospitals | 1.0 |  |
| High-volume hospitals | 0.77(0.62-0.95) * | 0.017 |
| Surgery type |  |  |
| Clipping | 1.0 |  |
| Coiling | 0.99(0.79-1.23) | 0.896 |
| Severity |  |  |
| mild | 1.0 |  |
| severe | 14.01(10.58-18.56) * | <.001 |
| Age |  |  |
| 18-45 | 1.0 |  |
| 46-59 | 1.26(0.93-1.72) | 0.140 |
| 60-69 | 1.70(1.12-2.44) * | 0.004 |
| ≥70 | 4.45(3.07-6.44) * | <.001 |
| Male, sex | 1.0 |  |
| Female, sex | 1.02(0.80-1.29) | 0.902 |
| Health insurance type |  |  |
| Health insurance | 1.0 |  |
| Medical aid | 2.26(1.35-3.78) * | 0.002 |
| Arrival mode |  |  |
| EMS | 1.0 |  |
| No EMS | 0.45(0.34-0.60) * | <.001 |
| Medical history |  |  |
| CCI score |  |  |
| 0 | 1.0 |  |
| 1 | 1.33(1.00-1.77) * | 0.046 |
| 2 | 1.47(1.06-2.03) * | 0.020 |
| ≥3 | 2.03(1.49-2.75) * | <.001 |

OR= odds ratio, CI = confidence interval, EMS=emergency medical services, CCI=charlson’s comorbidity index

* P < 0.05 significance.
