# Supplementary material for: Short- and long-term mortality of subarachnoid hemorrhage according to hospital volume and severity using a nationwide multicenter registry study
Source: Front Neurol. 2022 Aug 5;13:952794. doi: 10.3389/fneur.2022.952794 (PMC9389169; doi:10.3389/fneur.2022.952794)
Supplement: Supplementary file 3 [file Table_3.DOCX]

Supplementary Table 3. Cox analysis of death according to total 2,634 subarachnoid hemorrhage patients

|  | 3-month | | 1-year | | 2-year | | 4-year | |
| --- | --- | --- | --- | --- | --- | --- | --- | --- |
|  | HR(95% CI) | p-value | HR(95% CI) | p-value | HR(95% CI) | p-value | HR(95% CI) | p-value |
| Medical facility type |  |  |  |  |  |  |  |  |
| Low-volume hospitals | 1.0 |  | 1.0 |  |  |  |  |  |
| High-volume hospitals | 0.75(0.62-0.92) * | 0.005 | 0.83(0.68-1.00) | 0.055 | 0.89(0.74-1.08) | 0.249 | 0.96(0.75-1.23) | 0.750 |
| Surgery type |  |  |  |  |  |  |  |  |
| Clipping | 1.0 |  | 1.0 |  |  |  |  |  |
| Coiling | 0.93(0.76-1.14) | 0.479 | 1.01(0.83-1.24) | 0.922 | 0.98(0.80-1.19) | 0.805 | 1.03(0.81-1.32) | 0.805 |
| Clinical status |  |  |  |  |  |  |  |  |
| mild | 1.0 |  | 1.0 |  | 1.0 |  | 1.0 |  |
| severe | 1.81(1.44-2.27) * | <.001 | 1.58(1.26-1.96) * | <.001 | 2.05(1.65-2.55) * | <.001 | 2.50(1.90-3.28) * | <.001 |
| Age |  |  |  |  |  |  |  |  |
| 18-45 | 1.0 |  | 1.0 |  | 1.0 |  | 1.0 |  |
| 46-59 | 1.33(0.95-1.87) | 0.099 | 1.12(0.79-1.57) | 0.525 | 1.22(0.87-1.70) | 0.258 | 0.92(0.61-1.41) | 0.712 |
| 60-69 | 1.55(1.06-2.28) | 0.247 | 1.03(0.70-1.51) | 0.877 | 0.99(0.68-1.44) | 0.936 | 1.13(0.71-1.80) | 0.598 |
| ≥70 | 1.91(1.32-2.75) * | <.001 | 1.13(0.78-1.63) | 0.533 | 1.03(0.72-1.48) | 0.863 | 1.05(0.67-1.64) | 0.832 |
| Male, sex | 1.0 |  | 1.0 |  | 1.0 |  | 1.0 |  |
| Female, sex | 0.83(0.66-1.04) | 0.102 | 1.10(0.88-1.38) | 0.406 | 1.20(0.96-1.50) | 0.113 | 1.11(0.84-1.47) | 0.472 |
| Health insurance type |  |  |  |  |  |  |  |  |
| Health insurance | 1.0 |  | 1.0 |  | 1.0 |  | 1.0 |  |
| Medical aid | 1.00(0.67-1.49) | 0.990 | 1.34(0.90-1.99) | 0.105 | 1.09(0.73-1.62) | 0.689 | 1.44(0.88-2.34) | 0.147 |
| Arrival mode |  |  |  |  |  |  |  |  |
| EMS | 1.0 |  | 1.0 |  | 1.0 |  | 1.0 |  |
| No EMS | 0.71(0.50-1.00) * | 0.048 | 0.83(0.59-1.17) | 0.288 | 0.73(0.52-1.02) | 0.066 | 0.96(0.66-1.39) | 0.824 |
| Medical history |  |  |  |  |  |  |  |  |
| CCI score |  |  |  |  |  |  |  |  |
| 0 | 1.0 |  | 1.0 |  | 1.0 |  | 1.0 |  |
| 1 | 0.90(0.68-1.19) | 0.454 | 0.82(0.62-1.08) | 0.157 | 0.98(0.74-1.30) | 0.898 | 0.95(0.68-1.35) | 0.792 |
| 2 | 0.92(0.69-1.23) | 0.573 | 0.79(0.58-1.06) | 0.109 | 0.81(0.60-1.09) | 0.161 | 0.72(0.50-1.05) | 0.088 |
| ≥3 | 0.86(0.65-1.14) | 0.295 | 0.54(0.41-0.73) * | <.001 | 0.62(0.47-0.83) * | 0.001 | 0.60(0.42-0.85) * | 0.004 |

Values are presented as HR (95% CI), HR=Hazard Ratio, 95% CI=95% confidence intervals.

Mild severity=NIHSS≤15, GCS ≥ 10

Severe severity=NIHSS≥16, GCS ≤ 9

EMS=emergency medical services, CCI=Charlson comorbidity index

* P < 0.05 significance.
