# Supplementary figures and images for: Short- and long-term mortality of subarachnoid hemorrhage according to hospital volume and severity using a nationwide multicenter registry study
Source: Front Neurol. 2022 Aug 5;13:952794. doi: 10.3389/fneur.2022.952794 (PMC9389169; doi:10.3389/fneur.2022.952794)

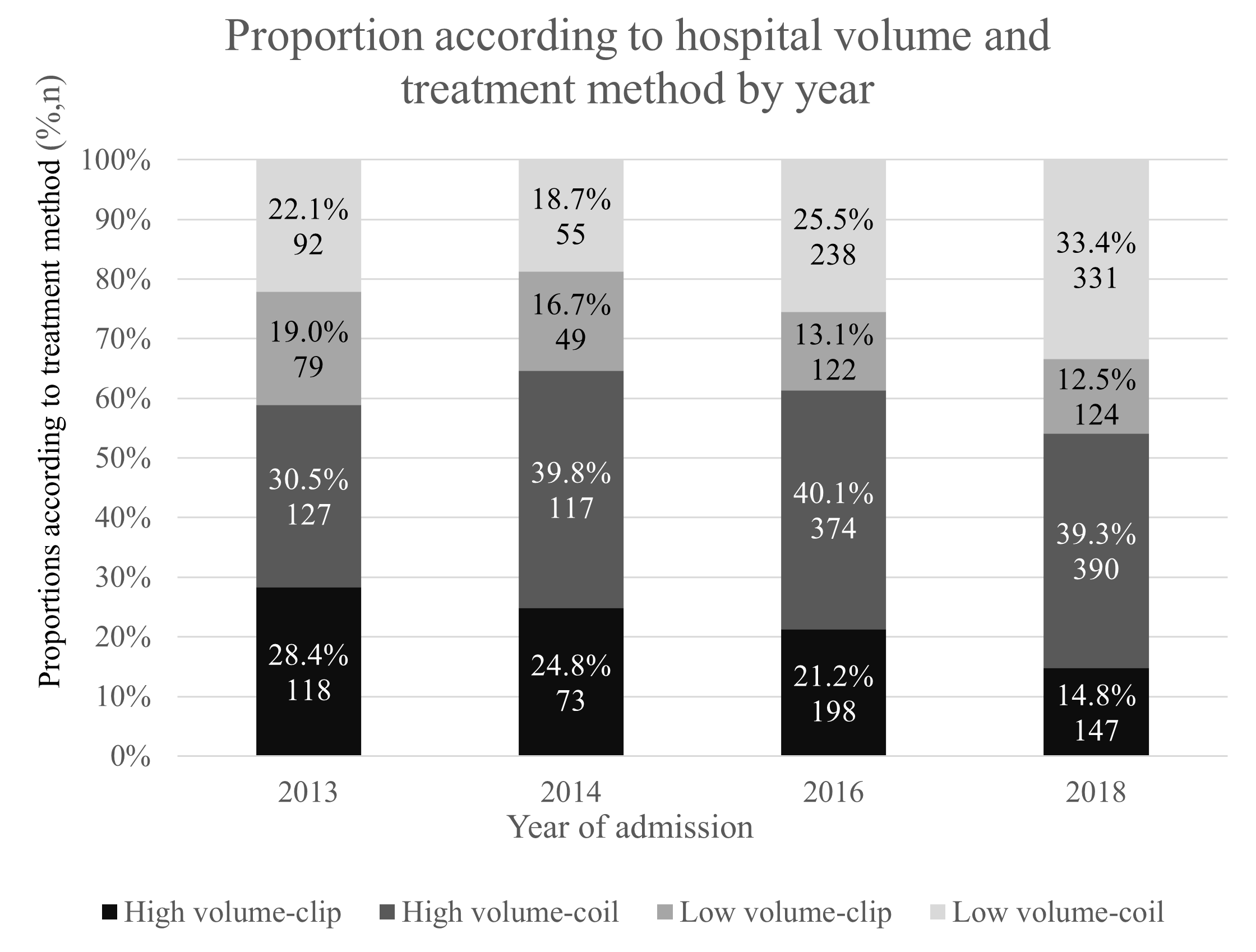

Supplement: Supplementary file 4 [file Image_1.TIF]

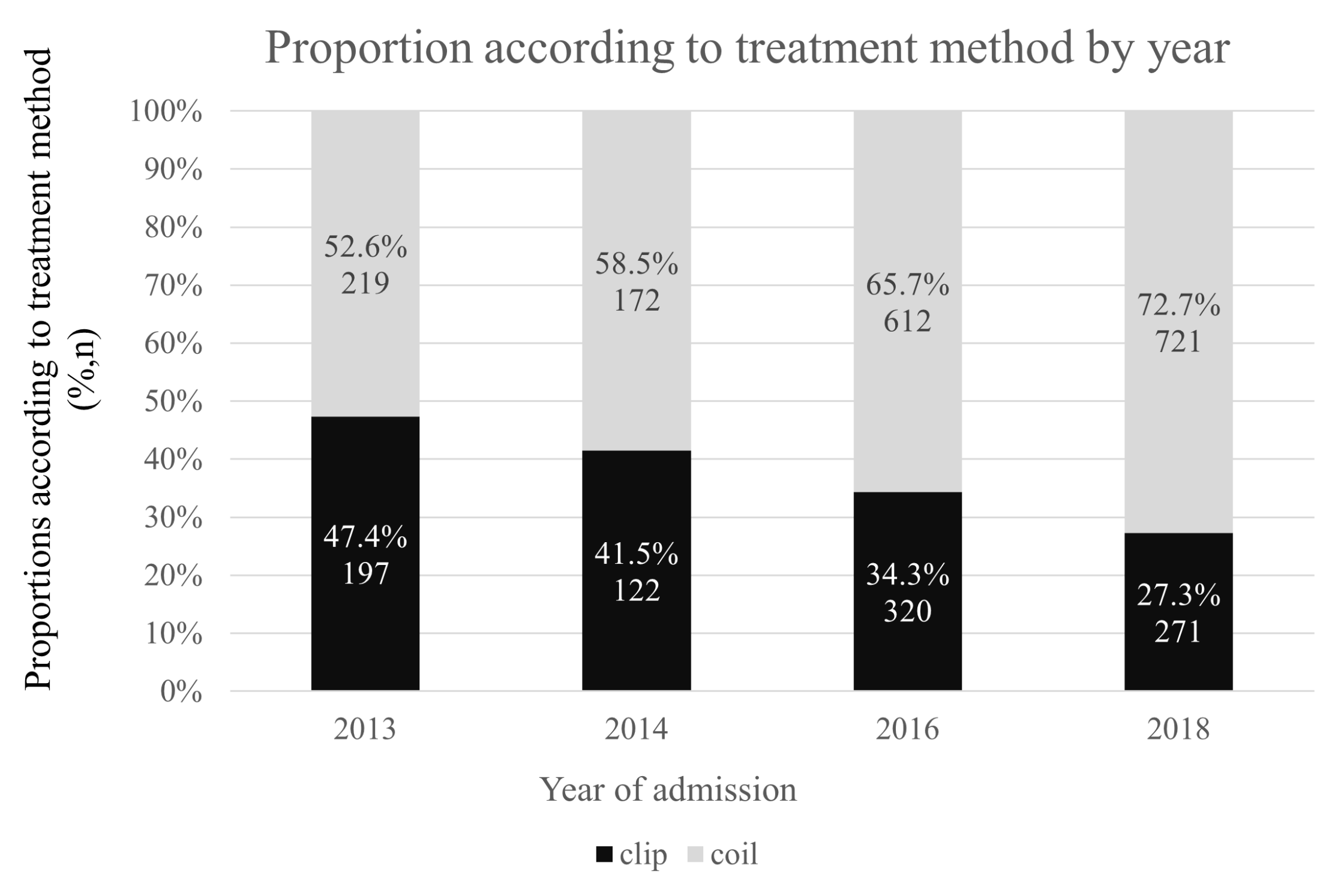

Supplement: Supplementary file 5 [file Image_2.TIF]
